# Children systemic lupus erythematosus-associated pancreatitis

**DOI:** 10.1186/s13075-024-03265-1

**Published:** 2024-01-17

**Authors:** Dan Zhang, Jianming Lai, Gaixiu Su, Jia Zhu, Min, Kang, Ming Li, Yingjie Xu, Li Meng

**Affiliations:** 1 Department of Rheumatology and Immunology, Children’s Hospital of Capital Institute of Paediatrics, No.2 Yabao Road, Chaoyang District, Beijing, 100020 China; 2 Department of Internal Medicine, Children’s Hospital of Capital Institute of Paediatrics, Beijing, China

## Abstract

**Objective:**

To early recognise and improve the prognosis of children systemic lupus erythematosus (cSLE)-associated pancreatitis by summarising and analysing clinical features and prognosis data from 12 cases.

**Methods:**

Retrospective analysis of clinical data from 12 cases of cSLE-associated pancreatitis diagnosed and treated from January 2016 to December 2021 at hospitals such as Children’s Hospital of Capital Institute of Paediatrics.

**Results:**

The median SLEDAI-2K score for disease activity was 18.00 (range 12.25–21.00) in the case group and 10.00 (range 7.00–18.00) in the control group, with a statistically significant difference (*P* < 0.05) between the two groups. The case group had a higher proportion of abdominal pain, vomiting, abdominal distension, pleural effusion, Raynaud’s phenomenon (RP), splenic infarction, and concurrent macrophage activation syndrome (MAS) than the control group, with a statistically significant difference (*P* < 0.05). Serum ferritin (SF), alanine transaminase (ALT), aspartate transaminase (AST), lactate dehydrogenase (LDH), amylase, and increased 24-h urine protein levels were statistically different between the two groups (*P* < 0.05); platelet counts (PLT) reduction was also statistically different (*P* < 0.05). The case group had a higher proportion of methylprednisolone pulse therapy, cyclophosphamide pulse therapy during remission induction, and therapeutic plasma exchange than the control group, with a statistically significant difference (*P* < 0.05) between the two.

**Conclusion:**

CSLE-associated pancreatitis has a high fatality rate. The presence of RP, splenic infarction, pleural effusion, and MAS warrants attention from clinicians regarding the possibility of pancreatitis. Once pancreatitis is detected, the primary disease needs active treatment for better prognosis.

## Introduction

Children systemic lupus erythematosus (cSLE) refers to SLE that occurs in childhood. It is an autoimmune disease of unknown aetiology that can involve multisystem organs. Gastrointestinal tract involvement is common in cSLE, with an incidence of 19% [1–4]. But lupus-associated pancreatitis is rare, with an incidence of only 0.7–4% [5] and can be life-threatening in severe cases. There is no large-sample clinical study on cSLE-associated pancreatitis. This study analysed the clinical characteristics and prognosis data of 12 cases of cSLE-associated pancreatitis, compares them with cSLE without pancreatitis, and explores the aetiology and risk factors of cSLE-associated pancreatitis for early identification and better prognosis.

## Methods

### Study design

In a retrospective case-control study, 12 children with cSLE and pancreatitis were selected as the case group. They were diagnosed and treated at the Department of Rheumatology and Immunology, Children’s Hospital of Capital Institute of Paediatrics (CIP), the Department of Paediatrics, The Second Hospital of Hebei Medical University, the Department of Paediatric Nephrology and Rheumatology, Shengjing Hospital of China Medical University, the Department of Rheumatology and Immunology, etc., from January 2016 to December 2022. A control group was randomly selected from children with cSLE but without pancreatitis who were diagnosed and treated during the same period at the Department of Rheumatology and Immunology, Children’s Hospital of Capital Institute of Paediatrics. Cases in the control group were matched with the age and gender of the case group in a 1:4 sample-size ratio of case group to control group. Exclusion criteria included the following diseases: (1) tumours; (2) inherited metabolic disorders; (3) pre-existing pancreatic and gallbladder diseases; and (4) other pre-existing chronic diseases, such as hypertension, diabetes mellitus. CSLE diagnosis had to comply with the 1997 European League Against Rheumatism/American College of Rheumatology (EULAR/ACR) SLE diagnostic criteria [6]. This study was approved by the CIP Ethics Committee, which waived informed consent.

#### Data collection

Collect clinical manifestations, laboratory tests, imaging tests, treatment history, and follow-up information of the study subjects to summarise the clinical characteristics. Main observation indicators are as follows: (1) clinical symptoms: fever, gastrointestinal damage (nausea/vomiting/abdominal pain/diarrhoea), skin damage, arthritis, serositis, renal damage, neurological damage, etc.; (2) laboratory tests: complete blood count, urinalysis, C-reactive protein (CRP), dynamic erythrocyte sedimentation rate (ESR), serum ferritin (SF), biochemistry, coagulation, complement C3 and C4, hemodiastase, urine amylase, spectrum of antinuclear antibodies, spectrum of anti-neutrophil cytoplasmic antibodies, spectrum of antiphospholipid antibodies, anti-globulin test; (3) imaging tests: cardiac ultrasound, chest CT scan, abdominal ultrasound, contrast-enhanced abdominal CT scan; (4) SLEDAI score; (5) treatment and prognosis.

#### Relevant diagnostic criteria and disease activity index

Diagnostic criteria for pancreatitis are as follows: (1) persistent upper abdominal pain; (2) serum amylase and/or lipase three times the upper limit of normal range; (3) abdominal imaging examination shows changes consistent with the imaging of acute pancreatitis. Diagnosis can be made if two out of the three criteria are met. Macrophage activation syndrome (MAS) conforms to the 2009 preliminary diagnostic guidelines for SLE-MAS in children [7]. The Systemic Lupus Erythematosus Disease Activity Index-2000 (SLEDAI-2K) was used to assess cSLE disease activity: a score under four indicates inactive disease, while a score above five suggests disease activity, with higher scores indicating higher disease activity [8].

### Statistical analysis

Data processing was performed using IBM SPSS Statistics 26.0. A *P*-value of < 0.05 (two-tailed) was considered statistically significant. We first analysed measurement data by Kolmogorov-Smirnov test to see whether the data follows a normal distribution. Normally distributed continuous variables are expressed as mean ± standard deviation (mean ± SD), using the *t*-test for two-group comparison. Non-normally distributed continuous variables are expressed as the median (*M*) [interquartile range (IQR) p25–p75], using the rank-sum test for two-group comparison. Categorical data were expressed as frequency (*n*) and percentage (%), using the chi-squared (*χ*^2^) test for two-group comparison.

## Results

### General information

The case group comprised 12 female children aged 9–17, with the median age of onset at 13.00 (range 11.25–14.00). The median duration of cSLE-associated pancreatitis at diagnosis was 0.83 (range 0.37–10.00) months. The control group included 48 children aged 9–17, among whom were eight males and 40 females with a ratio of 1:5. The median age of onset was 13.00 (range 11.25–14.00). The median duration of cSLE at diagnosis was 1.00 (0.50–3.00) month. There were no statistically significant differences in gender and age of onset between the two groups. The median SLEDAI-2K score for disease activity was 18.00 (range 12.25–21.00) in the case group and 10.00 (range 7.00–18.00) in the control group, with a statistically significant difference (*P* < 0.05) between the two groups.

### Clinical manifestations

Clinical manifestations between the two groups are shown in Table 1. Three cases in the case group were diagnosed as MAS due to persistent fever, hepatosplenomegaly, thrombocytopenia, elevated SF, and ALT/AST. The case group had a higher proportion of abdominal pain, vomiting, abdominal distension, pleural effusion, RP, splenic infarction, and concurrent MAS than the control group, with a statistically significant difference (*P* < 0.05) between the two groups.

**Table 1:** Univariate analysis of clinical manifestations in control and case groups

**Variables**	**SLE with pancreatitis (*N* = 12)**	**SLE without pancreatitis (*N* = 48)**	***P***
Gender (male), *n* (%)	0 (zero)	8 (16.7 %)	0.296
Age, year, median (IQR)	13.00 (11.25, 14.00)	13.00 (11.25, 14.00)	1.000
SLEDA-2K rating	18.00 (12.25, 21.00)	10.00 (7.00, 18.00)	**0.034**
Fever (yes), *n* (%)	8 (66.7 %)	25 (52.1%)	0.364
Abdominal pain, *n* (%)	12 (100 %)	1 (2.1 %)	< 0.001
Vomiting, *n* (%)	12 (100 %)	0 (0 %)	< 0.001
Abdominal bloating, *n* (%)	4 (33.3 %)	0 (0 %)	< 0.001
Diarrhoea, *n* (%)	0 (0 %)	1 (2.1 %)	1.000
Mouth ulcers, *n* (%)	2 (16.7 %)	10 (20.8%)	1.000
Rash (yes), *n* (%)	4 (33.3 %)	24 (50.0 %)	0.301
RP (yes), *n* (%)	2 (16.7 %)	0 (0 %)	0.037
Pleural effusion (yes), *n* (%)	5 (41.7 %)	5 (10.4 %)	0.030
Splenic infarction (yes), *n* (%)	2 (16.7 %)	0 (0 %)	0.037
Renal involvement (yes), *n* (%)	10 (83.3 %)	25 (52.1 %)	0.050
Arthritis, *n* (%)	1 (8.3 %)	8 (16.7 %)	0.786
Neurologic involvement, *n* (%)	5 (41.7 %)	7 (14.6 %)	0.09
Acute pericarditis, *n* (%)	1 (8.33 %)	1 (2.08 %)	0.281
MAS	3 (25 %)	0 (0 %)	< 0.001

*N* Number of patients in the group, *n* Number of cases, *SLE* Systemic lupus erythematosus, *IQR* Interquartile range, *SLEDA-2K* Systemic Lupus Erythematosus Disease Activity Index-2000, *RP* Raynaud’s phenomenon, *MAS* Macrophage activation syndrome

### Laboratory tests

Laboratory results between the two groups are shown in Table 2. SF, ALT, AST, LDH, amylase, and increased 24-h urine protein levels were statistically different between the two groups (*P* < 0.05); platelet count (PLT) reduction was statistically different between the two groups (*P* < 0.05).

**Table 2:** Univariate analysis of laboratory tests in control and case groups

**Variables**	**SLE with pancreatitis (*N* = 12)**	**SLE without pancreatitis (*N* = 48)**	***P***
ESR, median (IQR), mm/h	41.57 (29.50, 54.75)	30.00 (20.50, 48.75)	0.385
CRP, median (IQR), mg/l	3.82 (0.83, 17.71)	2.05 (1.00, 3.23)	0.705
SF, median (IQR), ng/dl	1160.66 (922.32, 1790.16)	190.42 (66.00, 530.25)	***0.000***
WBC, median (IQR), ×10^12^ /l	5.49 (2.54, 6.55)	4.81 (3.44, 6.82)	0.993
NEU, median (IQR), ×10^12^ /l	3.56 (1.67, 6.35)	2.90 (1.72, 4.17)	0.314
LYM, median (IQR), ×10^12^ /l	0.97 (0.81, 1.55)	1.33 (0.98, 2.15)	0.050
PLT, median (IQR), ×10^9^ /l	139.50 (80.50, 183.50)	219.50 (130.75, 261.25)	***0.029***
HGB, median (IQR), g/l	102.50 (91.50, 78.10)	110.00 (98.00,121.00)	0.189
ALT, median (IQR), u/l	117.50 (97.68, 478.10)	23.15 (16.10, 56.78)	***0.000***
AST, median (IQR), u/l	578.35 (178.43, 1166.88)	30.20 (22.13, 52.58)	***0.000***
HBDH, median (IQR), u/l	246.68 (246.68, 318.92)	210.50 (165.75, 265.75)	0.052
LDH, median (IQR), u/l	640.60 (562.50, 640.60)	269.50 (211.25, 404.50)	***0.001***
Blood amylase, median (IQR), u/l	405.20 (197.75, 875.65)	71.00 (50.25, 97.75)	***0.000***
ALB, median (IQR), u/l	36.24 (28.93, 39.51)	36.77 (33.83, 41.25)	0.345
BUN, median (IQR), mmol/L	5.48 (4.23, 7.21)	4.16 (3.21, 5.45)	0.065
SCr, median (IQR), μmol/l	56.84 (39.68, 58.29)	41.05 (36.48, 51.88)	0.091
24-h urine protein (IQR), μmol/l	1005.50 (521.00, 1415.37)	302.90 (102.87, 1005.50)	***0.013***
C3, median (IQR), g/l	0.30 (0.16, 1.08)	0.50 (0.37, 0.79)	0.155
C4, median (IQR), g/l	0.05 (0.02, 0.21)	0.06 (0.03, 0.14)	0.416
IgG ACA (positive), *n* (%)	3 (25 %)	8 (16.7 %)	0.802
IgM ACA (positive), *n* (%)	3 (25 %)	11 (22.9 %)	1.000
Anti-Ro antibodies (positive), *n* (%)	3 (25 %)	21 (43.8 %)	0.236
Anti-La antibodies (positive), *n* (%)	0	7 (15.6 %)	0.159
Anti-Sm antibodies (positive), *n* (%)	3 (25 %)	13 (27.1 %)	0.884
Anti-dsDNA antibodies (positive), *n* (%)	11 (91.7 %)	33 (68.8 %)	0.108
Coombs test (positive), *n* (%)	5 (41.7 %)	34 (70.8 %)	0.120

*N* Number of patients in the group, *n* Number of cases, *SLE* Systemic lupus erythematosus, *ESR* Erythrocyte sedimentation rate, *IQR* Interquartile range, *CRP* C-reactive protein, *SF* Serum ferritin, *WBC* White blood count, *NEU* Neutrophil, *LYM* Lymphocyte, *PLT* Platelet counts, *HGB* Haemoglobin, *ALT* Alanine transaminase, *AST* Aspartate transaminase, *HBDH* Hydroxybutyrate dehydrogenase, *LDH* Lactate dehydrogenase, *ALB* Albumin, *BUN* Blood urea nitrogen, *SCr* Serum creatinine, *C3* Complement component 3, *C4* Complement component 4, *IgG ACA* Anti-cardiolipin antibody IgG, *IgM ACA* Anti-cardiolipin antibody IgM

### Treatment

#### General treatment

All 12 children in the case group who developed pancreatitis were given symptomatic treatment with water fasting, intravenous fluid replacement, and inhibition of gastric acid and pancreatic enzyme secretion.

#### Surgical treatment

Among the 12 children in the case group with pancreatitis, two underwent exploratory laparotomy (16.7%). During surgery, we incised the pancreatic capsule and performed abdominal lavage. Drainage tubes were placed postoperatively.

Medication for the two groups is shown in Table 3. Methylprednisolone pulse therapy, cyclophosphamide pulse therapy during remission induction, and therapeutic plasma exchange were statistically different between the two groups (*P* < 0.05).

**Table 3:** Univariate analysis of treatment in case and control groups

**Variables**	**SLE with pancreatitis (*N* = 12)**	**SLE without pancreatitis (*N* = 48)**	***P***
Steroid, *n* (%)	12 (100 %)	47 (97.9 %)	0.614
Methylprednisolone pulse during remission induction, *n* (%)	11 (91.7 %)	5 (10.4 %)	< 0.001
Adequate methylprednisolone/prednisolone during remission induction	1 (8.3 %)	42 (87.5 %)	< 0.001
Immunosuppressant			
Hydroxychloroquine, *n* (%)	12 (83.3 %)	43 (89.6 %)	0.243
Cyclophosphamide during induction, *n* (%)	10 (83.3 %)	16 (33.3 %)	0.002
Mycophenolate mofetil during induction, *n* (%)	0 (0 %)	19 (39.6 %)	0.008
Mycophenolate mofetil during maintenance, *n* (%)	7 (83.3 %)	16 (33.3 %)	0.111
Methotrexate, *n* (%)	0 (0 %)	5 (10.4 %)	0.243
Tacrolimus, *n* (%)	0 (0 %)	4 (8.3 %)	0.301
Cyclosporine, *n* (%)	1 (8.3 %)	2 (4.2 %)	0.605
Leflunomide, *n* (%)	0 (0 %)	2 (4.2 %)	0.472
Belimumab, *n* (%)	1 (8.3 %)	1 (2.1 %,)	0.281
Rituximab, *n* (%)	1 (8.3 %)	6 (12.5 %)	0.688
Plasma exchange, *n* (%)	2 (16.7 %)	0 (0 %)	0.004

*N* Number of patients in the group, *n* Number of cases, *SLE* Systemic lupus erythematosus

### Prognosis and outcome

Prognosis and outcome between the two groups are shown in Table 4. Twelve children in the case group were followed up for 1–7 years. Within 1 week to 2 months of disease duration, eight of them recovered completely from pancreatitis, with amylase levels returning to normal and remission in SLE. Among them, case 3 had high disease activity and developed secondary MAS. The case transiently improved after aggressive treatment, experienced severe infections during the treatment of primary disease, and saw her MAS worsened. The family gave up treatment. Case 8 had high disease activity, refused surgery after developing pancreatitis complicated by peritonitis, and eventually died. Cases 9 and 12 had high disease activity with presence of multi-organ failure and MAS. They both died.

**Table 4:** Prognosis and outcome in case groups

**Patients**	**Time of Serum amylase return to normal**	**Dead or alive**	**Cause of death**
1	7 days	Alive	Uninvolved
2	14 days	Alive	Uninvolved
3	Uninvolved	Dead	MAS
4	1 month	Alive	Uninvolved
5	2 months	Alive	Uninvolved
6	9 days	Alive	Uninvolved
7	12 days	Alive	Uninvolved
8	Uninvolved	Dead	Pancreatitis
9	Uninvolved	Dead	Multi-organ failure and MAS
10	15 days	Alive	Uninvolved
11	1.5 months	Alive	Uninvolved
12	Uninvolved	Dead	Multi-organ failure and MAS

*MAS* Macrophage activation syndrome

## Discussion

This is a multicentre clinical study of cSLE-associated pancreatitis in China. Given the disease’s low incidence in children [5] and previous domestic reports of it were all on isolated cases, this study represents the largest number of cases included in the case group of its kind in China. First, the study results reveal that the SLEDAI-2K score at the onset of disease in the case group was 18, higher than that of the control group, indicating that cSLE-associated pancreatitis is a critical complication of cSLE. The fatality rate in the case group was as high as 33.3%, similar to the reported rate of 37.04% in the literature [6]. Secondly, this study found that cSLE-associated pancreatitis is more likely to present with RP, splenic infarction, pleural effusion, and concurrent MAS. Thirdly, abdominal pain of varying levels was observed in all cases of the case group (100%), significantly more than in the control group, indicating that the occurrence of pancreatitis should be considered in cSLE patients presenting with acute abdominal pain. Fourthly, this study also found higher levels of 24-h urine protein, SF, ALT, AST, LDH, and amylase in the case group. Therefore, clinicians should closely monitor abdominal symptoms in cSLE cases. When lupus pancreatitis is suspected, renal function, SF, liver enzymes, and amylase should be assessed more attentively to monitor the disease progression. Lastly, during induction, the case group received methylprednisolone pulse therapy, cyclophosphamide pulse therapy, and therapeutic plasma exchange more frequently than the control group. This suggests that for early detection of pancreatitis, high-intensity treatment regimen should be chosen as mentioned above. In the presence of surgical indications, active surgical intervention should be administered to gain more time for remission of the primary disease of SLE.

The pathogenesis of SLE-associated pancreatitis remains unclear, but vascular trauma is one of the main causes [6, 9–14]. Vascular trauma includes necrotising vasculitis, arterial thromboembolism caused by severe hypertension and antiphospholipid syndrome, vascular endothelial injury, immune complex deposition, and complement activation in pancreatic arterial walls, among others. The mechanism by which RP occurs with SLE is not entirely clear, but endothelial dysfunction might underlie its pathophysiology. It is currently believed to be caused by increased endothelin, decreased calcitonin gene-related peptide, the presence of anti-fibrin-binding tissue-type plasminogen activator (t-PA) in circulating blood, decreased fibrinolytic activity in the organism, and coagulation in small blood vessels. In addition, increased protein tyrosine kinase (PTK) activity in vascular endothelial cells might be associated with RP development [6], suggesting that vascular endothelial injury could be a mechanism leading to RP in SLE-associated pancreatitis. Literature reports suggest that splenic infarction in SLE is associated with the deposition of circulating immune complexes on vascular walls and the activation of the complement pathway by the circulating immune complexes; both the deposition and the activation stimulate vascular endothelial injury [14–16], potentially explaining the higher proportion of splenic infarction cases in the study’s case group compared to the control group. Lupus anticoagulant and antiphospholipid antibodies are risk factors for arteriovenous thrombosis or embolism in SLE, and these mechanisms are also considered to be the pathogenesis of SLE-associated pancreatitis [17].

Risk factors for cSLE-associated pancreatitis remain unclear. In this study, one child in the case group exhibited fever, neurological damage, intravascular haemolytic anaemia, thrombocytopenia, and renal failure, combined with pancreatitis and heart failure. The patient tested positive for ADAMTS-13 antibodies and was diagnosed with thrombotic thrombocytopenic purpura (TTP). Thus, TTP may be a high-risk factor for cSLE-associated pancreatitis. Among the four deceased cases in the case group, three (25%) had MAS, a severe, acute and lethal syndrome. In 2009, Parod et al. proposed preliminary diagnostic guidelines for childhood SLE-MAS through statistical analysis of laboratory indicators of SLE-MAS [18]. In this study, the proportion of MAS cases in the case group was higher than in the control group (*P* < 0.05). Therefore, in cSLE cases presenting clinical features that are difficult to be explained by the primary disease activity, such as fever, bleeding, hepatosplenomegaly, lymphadenectasis, neurological dysfunction, and heart and renal failure, the diagnosis of SLE-associated MAS is considered, particularly when SF, AST, and LDH are consistently elevated, and dynamic erythrocyte sedimentation rate decreases. Further attention should be given to the occurrence of pancreatitis.

Literature search did not reveal any correlation between pleural effusion and the occurrence of pancreatitis in SLE patients. In this study, 24-h urine protein levels were significantly higher in the case group than in the control group. Thus, whether the occurrence of pleural effusion is caused by renal protein loss requires further research. Currently, there is limited research on cSLE-associated pancreatitis at home and abroad, and there is a lack of large-sample data. Although this study is the multicentre study with the biggest number of cases in China at present, limitations still exist due to the small sample size.

## Conclusion

In conclusion, cSLE-associated pancreatitis has a high fatality rate. The presence of RP, splenic infarction, pleural effusion, and MAS warrants attention from clinicians regarding the possibility of pancreatitis. During remission induction, methylprednisolone pulse therapy, cyclophosphamide pulse therapy, and therapeutic plasma exchange are effective. In addition, TTP and MAS could be risk factors for cSLE-associated pancreatitis. Based on the conclusions of this study, we hope that clinicians can recognise cSLE-associated pancreatitis early for better prognosis and lower fatality rate.

## What does this mean for patients?

CSLE is an autoimmune disease of unknown aetiology that can involve multisystem organs. It can lead to gastrointestinal damage, with lupus-associated pancreatitis being a highly fatal complication. Therefore, early detection of the disease and active treatment are essential for improving the prognosis of patients with cSLE-associated pancreatitis.

In our study, we conducted a retrospective analysis of clinical data on 12 paediatric patients with cSLE-associated pancreatitis from several children’s hospitals in China over a 6-year period. We compared these cases with 48 cSLE patients without pancreatitis who were treated at the Rheumatology Department, Children’s Hospital of Capital Institute of Paediatrics, during the same period. Results reveal that abdominal pain, vomiting, abdominal distension, pleural effusion, RP, and splenic infarction were the primary clinical symptoms of cSLE-associated pancreatitis. Once this condition is suspected clinically, it is necessary to closely monitor SF, ALT, AST, LDH, amylase, and 24-h urine protein. And aggressive treatments such as glucocorticoid pulse therapy and cyclophosphamide pulse therapy are recommended for better prognosis.

## Data Availability

None.

## Abbreviations

cSLE: Children systemic lupus erythematosus
MAS: Macrophage activation syndrome
SF: Serum ferritin
ALT: Alanine transaminase
AST: Aspartate transaminase
LDH: Lactate dehydrogenase
PLT: Platelet counts
ESR: Dynamic erythrocyte sedimentation rate
SLEDAI-2K: SLE Disease Activity Index-2000
IQR: Interquartile range
RP: Raynaud’s phenomenon
t-PA: Tissue-type plasminogen activator
PTK: Protein tyrosine kinase
TTP: Thrombotic thrombocytopenic purpura
